# Rhythmic cued motor imagery and walking in people with multiple sclerosis: a randomised controlled feasibility study

**DOI:** 10.1186/s40814-015-0021-3

**Published:** 2015-07-11

**Authors:** Barbara Seebacher, Raija Kuisma, Angela Glynn, Thomas Berger

**Affiliations:** 1 University of Brighton, School of Health Sciences, Robert Dodd Building, 49 Darley Road, Eastbourne, BN20 7UR UK; 2 Clinical Department of Neurology, Medical University of Innsbruck, Anichstrasse 35, 6020 Innsbruck, Austria

**Keywords:** Multiple sclerosis, Physiotherapy, Rhythmic cued motor imagery, Walking

## Abstract

**Background:**

Novel physiotherapy approaches such as motor imagery and rhythmic auditory stimulation have been shown to improve walking in people with multiple sclerosis (MS). Rhythmic cued motor imagery was used in this study, whose objectives were to evaluate the feasibility of a larger randomised controlled trial (RCT) in people with MS and to obtain information on walking.

**Methods:**

Thirty adult people with MS who scored 1.5–4.5 on the Expanded Disability Status Scale were recruited at the MS Clinic Innsbruck, Austria. Participants were randomly allocated to one of three groups, all receiving usual care: 17 min of motor imagery, six times per week, for 4 weeks, with music (A) or metronome cues (B) and (C) controls. Primary outcomes were recruitment rates, retention, compliance, adverse events and fatigue (Modified Fatigue Impact Scale). Secondary outcomes were walking speed (Timed 25-Foot Walk) and walking distance (6-Minute Walk Test).

**Results:**

We achieved our recruitment target by recruiting 12 participants per month, a mean eligibility rate of 40.1 % (95 % confidence interval (CI) 35.8, 44.6 %) out of 2500 MS Centre patients, mean consent rate of 15.9 % (95 % CI 11.3, 21.7 %) plus 54.5 % (95 % CI 47.4, 61.4 %) of eligible patients who expressed their interest to participate. Retention of 100 %, no adverse events, good compliance, high acceptability of the interventions and no worsening of fatigue confirmed feasibility. The mean improvement in walking speed in both groups A and B was −0.9 s (95 % CI −1.3, −0.5), and mean worsening in group C was 0.4 s (95 % CI −0.3, 1.1). The mean improvement in walking distance in group A was 68.1 m (95 % CI 51.4, 84.7) and in group B 92.9 m (95 % CI 55.2, 130.5), and mean worsening in group C was −9.4 m (95 % CI −35.6, 16.9).

**Conclusions:**

Results from our study showed that a full-scale RCT is feasible to investigate the effects of rhythmic cued motor imagery on walking in people with MS, with no changes to the interventions and assessments. Based on the walking improvements, a total sample size of 138 participants was calculated. Stratified blocked randomisation, allocation concealment and blinding will be used in the main study.

**Trial registration:**

ISRCTN: ISRCTN67054113

## Background

Multiple sclerosis (MS) is an inflammatory demyelinating disease of the central nervous system affecting a person’s ability to walk, participation and quality of life [1]. Many people with MS adopt a sedentary lifestyle due to walking impairment [2] with 60 to over 90 % having walking problems [3]. In particular, reduced walking speed has a direct impact leading to reduced activities of daily living [3]. Physiotherapy has been shown to be effective in improving the individuals’ walking [4].

Recently, novel physiotherapy strategies, such as motor imagery and rhythmic auditory stimulation have been developed to improve walking speed and distance in people with neurologic disorders. Motor imagery is the mental execution of movements without any actual movement performance [5]. The advantage of motor imagery over actual movement practice is that it does not cause motor fatigue and the person is not at risk of falls as it can be practised while seated. Only one uncontrolled study was identified that investigated the effect of 5 weeks of motor imagery training on fatigue, walking and quality of life in 20 people with MS, with a median Expanded Disability Status Scale (EDSS) score of 2.0 (range 1.0, 6.0), corresponding with mild to moderate MS [6]. Results from their study were significant improvements in fatigue and quality of life, without effect sizes reported, but only trends for improvement in walking. To our knowledge, so far, no studies have investigated the effects of rhythmic cued motor imagery on walking performance in people with MS.

In people with other neurologic disorders, motor imagery has been shown to improve motor performance, in particular in people with stroke [7, 8], with moderate effect sizes [8]. Two different perspectives can be adopted during motor imagery, an internal, first-person (kinaesthetic mode) or an external, third-person (visual mode) perspective [5]. In the kinaesthetic mode, the person experiences/feels his or her own body moving, and in the visual mode, the person imagining watches himself or herself. For motor imagery of walking, individuals need to be ambulatory since there is a relationship between mental and physical execution of a movement [9]. It has been demonstrated that in people with motor impairment, the kinaesthetic mode is more effective than the visual mode because of a more efficient motor learning [10] due to sensory information [11]; therefore, the kinaesthetic imagery has been adopted for the current study.

Motor imagery can be practised with or without verbal guiding and additional, visual or auditory, cues [10]. Heremans and colleagues demonstrated that in people with moderate to severe MS, motor imagery ability correlated more with cognitive than physical impairment during an upper limb task [12] and was enhanced by rhythmic auditory cues [13]; therefore, rhythmic auditory cues were used in the current study.

Rhythmic auditory stimulation is another novel physiotherapy approach in walking rehabilitation which uses rhythmic auditory cues to synchronise the person’s steps with the external rhythm [14, 15]. It has been shown that intrinsically rhythmic movements such as walking are facilitated most effectively by even, rhythmic pulses [16]. Rhythmic auditory stimulation with metronome cues versus rhythmically accentuated music has been investigated in different populations. Studies have found that music but not metronome cues at the same tempo led to a significant increase in walking speed in healthy individuals [17, 18], with moderate effect sizes [17]. The authors suggested that additional auditory elements in the music may have facilitated walking more than simple metronome cues. Rhythmic auditory stimulation was shown to improve walking in people with neurologic disorders other than MS [19, 20, 16], with high effect sizes in people with Parkinson’s disease [20]. In people with MS, only two small studies investigated the effect of rhythmic auditory stimulation on walking [21, 22]. Baram and Miller [21] used a metronome-based auditory feedback device in 14 patients with a median EDSS score of 4.5 (range 4, 6) and corresponding to moderate MS, to measure effects on walking speed and gait parameters, in comparison to 11 healthy controls. Results showed a 12.84 % improvement in walking speed and an 8.30 % improvement in stride length, but effect sizes were not reported. Conklyn and colleagues’ [22] home-based pilot study in 10 persons with MS used music with embedded metronome cues and gait training, which resulted in significant improvement in percentage double support time with moderate effects sizes in the treatment group.

According to our knowledge and a recent meta-analysis [23], to date, no other randomised controlled trials have investigated the effect of motor imagery combined with rhythmic auditory stimulation on walking in people with MS, whereas this approach was applied in people with stroke. In their pilot study, Kim et al. found significantly improved walking performance in people with stroke (*n* = 15), in particular with kinaesthetic motor imagery when compared to visual motor imagery and with auditory step rhythm in comparison to without [24]. No effect sizes of the improvements were reported.

Recent work supports our basic suggestion that the connections between rhythmic auditory and motor processing, that is sensorimotor synchronisation to rhythmic auditory stimuli, may also apply to motor imagery [25]. Additionally, music has various other beneficial influences, such as its ability to improve mood, to increase the amount of work a person does while listening and to enhance motivation [26, 18] which might impact on walking performance and imagined walking. Listening to music instead of a metronome might be more pleasurable which might increase adherence to the motor imagery in our study, which is important as the intervention is home-based. However, some participants might be distracted by the music and feel more comfortable with the metronome. Although motor imagery does not cause physical fatigue, it might induce mental fatigue which needs to be tested. Therefore, a subtle emphasis was put on the beat, using simple and concise verbal cues to provide a more precise metrical structure which might facilitate rhythmic entrainment and attention in participants [14]. Verbal cues were used to prompt one specific component of imagined walking, like taking long strides [27]. This was the rationale for conducting a pilot study in which motor imagery with metronome or musical rhythm were compared with each other and against a control group.

The first aim of the study was to test the feasibility of a full-scale randomised controlled trial (RCT). The second aim of the study was to evaluate changes in fatigue caused by rhythmic cued motor imagery, as increased fatigue might be a barrier to the main study. The third aim of the study was to obtain information on walking speed and walking distance to be able to calculate the sample size for the main study.

## Methods

Prior to study commencement, an MS advisory group selected from a self-help group had been consulted for advice, to clarify any questions, for example, in terms of their motor imagery ability or whether they had practised it previously, and their music preference, but also on the question of how much time they would spend on practising. Comments from the advisory group members were very valuable to the researchers as they provided an insight into the patients’ perspectives. In response to this input, for group A, motivating music with a pleasing melody was selected, and for group B, metronome cues with different tone pitches were chosen.

The study was a three-group parallel randomised controlled single-centre pilot trial to provide information for a subsequent larger randomised controlled trial. Therefore, the sample size was based on the specific objectives of the study such as feasibility, attrition, adverse events and calculation of the sample size for the main study rather than on the estimated effects of the interventions.

The pilot study involved 30 people with MS and compared participants in three equally sized groups: motor imagery with rhythmically accentuated music and verbal cueing (group A), motor imagery with metronome cues and verbal cueing (group B) and a control group (group C). Participants in all groups received usual care and weekly phone calls. The study was conducted as follows: Initial visit to clinic for baseline assessment and instruction and provision of compact disc (CD), home-based intervention, then participants were contacted by phone once weekly to support the use of CD and to address any study related questions. Participants returned for reassessment after completing the 4-week intervention. Blinding was not possible as one physiotherapist (BS) was conducting the intervention instructions and assessments, and the participants became aware whether they were in an intervention or control arm. The measurements were taken at the MS Clinic of the Clinical Department of Neurology, Medical University of Innsbruck, Austria.

### Ethic statement

The study was approved by the Faculty Research Ethics Governance Committee of the University of Brighton on 9 January 2014 (reference number 13 053) and the Ethics Committee of the Medical University of Innsbruck on 2 April 2014 (reference number AN2014-0052 334/4.14). All participants provided written informed consent. Participants were reimbursed for travel expenses only.

### Participants and recruitment

**Fig. 1:**
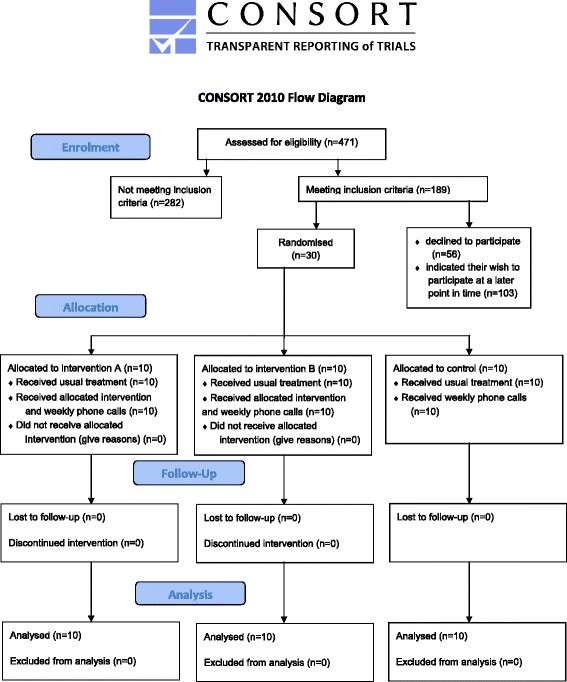
CONSORT flow diagram

Recruitment used unselected consecutive sampling and restricted randomisation from 9 April to 16 June 2014. A CONSORT flow diagram is shown in Fig. 1.

Inclusion criteria were people with mild to moderate MS (Expanded Disability Status Scale, EDSS 1.5 to 4.5) [28], aged 18 years or over, MS according to McDonald’s criteria [29], all MS phenotypes, any ethnicity, German speaking (questionnaires, instructions).

Exclusion criteria were concomitant other diseases which may affect rhythmic cued motor imagery or walking (e.g. untreated hearing impairment), a relapse of MS within the last 3 months, recent change of medication which is known to affect walking within the last 2 months, known pregnancy, overt symptoms or signs of depression or cognitive dysfunction, diagnosed and documented by Innsbruck MS Clinics. A relapse during the intervention period led to exclusion of the participant.

Participants were allocated randomly by drawing sealed envelopes from a black bag, with letters ‘A’, ‘B’ or ‘C’ which were consistent with the three study groups. The researchers aimed at 10 participants in each group so that the randomisation was restricted insofar as the bag contained only 30 envelopes. Within this procedure, concealment of allocation has become redundant as the physiotherapist who performed the instructions and measures could not influence group allocation.

### Data collection

Demographic (gender, age) and MS disease specific data (current EDSS) were extracted from patients’ charts; study specific assessment data were collected by one physiotherapist, thus avoiding any inter-rater variability. Adverse events were recorded during or immediately after a visualisation session. Withdrawals or other reasons for exclusion from the study were recorded. For measures at baseline and follow-up, the Modified Fatigue Impact Scale (MFIS) [30, 31], the Timed 25-Foot Walk (T25FW) [32] and the 6-Minute Walk Test (6MWT) [33] were used. Baseline and follow-up assessments were performed at the same time of the day, in view of daytime fluctuations in fatigue.

### Intervention

The intervention of this study consisted of rhythmic cued motor imagery, the cueing being provided either by instrumental music or by a metronome, both with additional rhythmic verbal cueing. Study participants were instructed how to use the rhythmic cued motor imagery as suggested in previous studies [34, 10]. First, participants were informed in lay language about the concept of motor imagery and its application and effects in sports and neurorehabilitation. The approach of rhythmic cued motor imagery was introduced. Secondly, participants learned about the two perspectives of motor imagery (internal, external), as in clinical practice people use both perspectives when asked to imagine a movement. Participants were given practical tips on kinaesthetic imagery of walking, that is, they were explained to ‘feel’ themselves walking during the imagery. Finally, for this study, the participants were asked to imagine themselves walking in various ways, such as pacing, walking with large steps, raising the forefoot during walking, walking fast or stamping, as described on the CDs prepared by the researcher for this study.

The music type and beat was selected based on a published summary of practical guidelines and recent publications [15]: With the metronome cues and music, rhythmic cueing was in 2/4 or 4/4 metre with strong ON (on beat 1 or beats 1 and 3) and OFF beat patterns, and it was emphasised by rhythmic verbal cues of the researcher (e.g. rhythmic speech ‘step-step’, ‘toe-off’) [15, 14]. Suitable rhythmical sequences were cut together and mixed with instructions on motor imagery of walking at various tempi and provided on the CD. The same procedure was applied for the metronome audio mix. According to the Austrian State Authorized Society of Authors, Composers and Publishers (AKM), no copyright permission was required as the music was used for research purposes only. Participants of all groups received their normal or no treatment. If participants needed new physiotherapy or drug interventions that could have an impact on walking, they would be withdrawn from the study.

At the baseline visit, participants in groups A and B received the CD, an introduction to motor imagery including a short practice and instructions from the researcher on the intervention task. They were asked to practise at home in a sitting position, with eyes closed and the kinaesthetic mode. Participants were asked to practise six times a week for 17 min for 4 weeks. Duration of both the practice and the study were based on the current literature on motor imagery, showing an average study duration of 34 days with a practice intensity of three times a week, for 17 min [10, 6]. The frequency of six times a week was chosen according to a recommendation by Peiris et al. [35] and a Cochrane review from Rietberg et al. [36].

Advice was given such as to practise at the best time of the day in terms of fatigue. Four audio mixes, each designed in the same way, were on one CD. After each week, the audio mix was changed with the intention to maintain attention with the motor imagery [15] and to facilitate adherence. The researcher called the participants every week, checking if they had any problems and supporting them in their use of motor imagery. Participants in the control group were also called weekly by the researcher to pay attention to them and asking for their health condition. Participants in all groups, including the control group, received their usual care which consisted of regular appointments with their physicians and MS specific immunomodulatory and symptomatic medication. The researcher is not a member of the MS Clinic team.

### Primary outcomes

The feasibility of conducting a larger main study with an equal or similar study design was tested and reported descriptively and narratively. These feasibility outcomes contained recruitment rates, retention rates, safety, compliance, participant acceptability of the study procedures, adverse events and fatigue. Monthly recruitment rates were recorded. Participant compliance with the interventions (kinaesthetic motor imagery, melodies, beats, tone pitches of the metronome cues, verbal cueing) and the acceptability thereof were recorded during weekly phone calls and at follow-up. Fatigue was assessed by the MFIS. Fatigue was measured to evaluate whether the interventions or assessments would over-fatigue participants. If so, the corresponding intervention would be dropped.

#### Modified fatigue impact scale

The MFIS is a modified form of the Fatigue Impact Scale [30] and one part of the Multiple Sclerosis Quality of Life Inventory (MSQLI) [31]. It is a 21-item Likert scale that evaluates, via self-report, the effects of fatigue on physical, cognitive and psychosocial functioning, with higher numbers indicating greater fatigue. The MFIS has an excellent reliability and moderate to high validity and responsiveness in people with MS [37, 38] and was recommended by the MS Outcome Measure Task Force [39], National MS Society [40] and European Committee for Treatment and Research in MS (ECTRIMS) Congress 2013 [41].

### Secondary outcomes

The secondary outcomes were walking speed as measured by the T25FW and walking distance as measured by the 6MWT. These measures were chosen for this study, because they are reliable, valid and sensitive to the expected changes in the study. They were recommended by the expert committees described above.

#### Timed 25-Foot Walk

The T25FW is the most commonly reported short walking test in people with MS. There is a consensus in the literature that a change of more than 20 % in walking speed corresponds to a clinically meaningful functional change in walking [42, 43]. The T25FW has excellent validity, reliability and responsiveness in people with MS [44, 45] and was administered according to instructions in the Multiple Sclerosis Functional Composite [46].

#### 6-Minute Walk Test

The 6MWT at maximum speed was used to measure walking distance. There is a discrepancy in the reporting of the clinically significant change for the 6MWT in people with MS which was an improvement of 10 % (21.6 m) as found by Baert et al. [47] and 20 % (88 m) as shown by Learmonth et al. in a study population more similar to that of the current study [43]. In the current study, ≥20 % improvement was considered clinically significant as a 20 % greater walking distance improves the individuals’ daily life [48]. The psychometric properties of the 6MWT have been found to be good to excellent in an MS population [33, 49]. It was carried out as recommended by the American Thoracic Society-Guidelines. Walking aids for both the T25FW and 6MWT were used if required but were documented and kept consistent during the two assessments.

### Statistical analysis

For the primary outcomes, the feasibility criteria were the recruitment rate and duration, retention rate, safety, adverse events, compliance, acceptability of the interventions and fatigue. The recruitment rate, consisting of the eligibility and consent rate, was calculated with 95 % CI according to Newcombe [50]. The success of recruitment was assessed (a) in view of a maximum possible duration of the pilot and main study of 22 months and (b) based on the potential to recruit the number of participants estimated by a sample size recalculation. The success of retention was reported as retention rate, the number of participants who fully participated in the study until follow-up and the percentage of missing data. Safety issues were monitored such as falls during the assessments. Adverse events, during and after the interventions, were reported. Severe adverse events would have led to early study termination. Compliance to the interventions was assessed during weekly phone calls and follow-up assessments. Participants reported the number of their rhythmic cued motor imagery practice per week, and descriptive statistics (median, range) was used to calculate compliance. Participant acceptability of the interventions was also assessed during phone calls and at follow-up assessments. Participants were asked how much they agreed to the kinaesthetic motor imagery and whether they had any problems with practice. They were asked about their agreement to the melodies and beats of the music and to the tone pitches of the metronome cues and the verbal cueing. Acceptability was reported narratively.

Only descriptive statistics was reported for the primary and secondary outcomes. Medians (range) were reported for ordinal data (fatigue), mean (95 % confidence interval (CI)) were reported for continuous data (walking speed and walking distance) and raw count (number, %) was reported for nominal data. Due to the nature of this feasibility study, it was decided not to conduct any efficacy statistical tests on the walking and fatigue data.

The sample size for the main study was calculated using Cohen’s d effect sizes [51] based on the walking outcomes. Cohen’s d was calculated using the formula *d* = (mean 1−mean 2)/corrected pooled standard deviation (SD). The corrected pooled SD for 80 % of trials was calculated using the formula (square root from ((SD1^2^ + SD2^2^)/2))∗1.293 [52]. Effect sizes were calculated with 95 % CI using an effect size calculator provided by the Centre for Evaluation and Monitoring at the University of Durham, UK. The sample size for the main study, based on effect sizes with 95 % CI, was calculated using the AI-Therapy online sample size calculator (AICBT Ltd, Oxford, UK). All statistics were performed using IBM SPSS software, release 22.0 (IBM Corporation, Armonk, NY, USA).

## Results

### Baseline characteristics

Participants’ baseline demographic data age and gender as well as disability in the three groups are shown in Table 1. Twenty-two females and 8 males were included in the study, representing a female-to-male ratio of 2.75:1, corresponding to previously reported data from the UK with 2.4:1 [53] and from Austria with 2.5:1 [54]. Due to the random allocation, more females were allocated to the intervention groups than males. There was no difference in age, disability (EDSS), MFIS total score, walking speed (T25FW) and walking distance (6MWT) between the groups at baseline. Baseline T25FW and 6MWT performances were consistent with results from other studies. None of the participants required a permanent walking aid; however, in group A, one participant used two walking sticks and one participant used one walking stick during all walking tests.

**Table 1:** Participants’ baseline characteristics

Parameter	Group A	Group B	Group C
	Music cued motor imagery	Metronome cued motor imagery	Control group
	*n* = 10	*n* = 10	*n* = 10
Females to males	10:0	7:3	5:5
Age (years)^a^	47.3 (38.4, 56.2)	41.8 (34.8, 48.8)	46.1 (39.8, 52.5)
EDSS^b^	3 (1.5, 4.5)	2.5 (1.5, 4.5)	2.5 (1.5, 4.0)
MFIS total score^b^	35 (3, 67)	32 (17, 50)	33.5 (0, 48)
Participants with fatigue (MFIS total score ≥38)	4/10	2/10	4/10
T25FW (s)^a^	6.1 (4.5, 7.6)	5.4 (4.5, 6.2)	5.2 (4.3, 6.1)
6MWT (m)^a^	453.1 (365.0, 541.1)	428.2 (352.8, 503.6)	484.7 (399.5, 569.8)

*EDSS* Expanded Disability Status Scale, *MFIS* Modified Fatigue Impact Scale, *T25FW* Timed 25-Foot Walk, *s* seconds, *6MWT* 6-Minute Walk Test, *m* metres

^a^Mean (95 % confidence interval)

^b^Median (range)

### Feasibility outcomes

#### Recruitment rate and retention rate

According to the research proposal and ethics approval, the maximum time for the pilot and main study is 22 months, given additional 4 months for data analysis and interpretation. Feasibility of the main study was assessed based on recruitment rates (Fig. 1). The MS Clinic Innsbruck is currently in care for approximately 2500 people with MS, and 471 were screened during recruitment (2 months) of those 189 were eligible. The sample size for the pilot study was *n* = 30. The eligibility rate was 40.1 % (95 % CI 35.8, 44.6 %). Of these 189 participants, 30 were enrolled to the study (15.9 %, with 95 % CI 11.3, 21.7 %), and further 103 eligible individuals declared their wish to participate in the study at a later point in time (54.5 %, with 95 % CI 47.4, 61.4 %). Therefore, it is expected that 1025 (95 % CI 895, 1115) out of 2500 MS Clinic patients are eligible and that 163 (95 % CI 116, 222) people with MS will participate in the main study. Due to the fact that additional 103 persons were willing to participate at a later point in time, the actual numbers might be even higher. The observed recruitment rate per month was 12 participants. All 30 participants completed the study, corresponding to a 100 % retention rate (95 % CI 88.6, 100 %). There were no missing data and no relapses of MS in this study; however, this cannot be expected for a larger trial. All participants were included in the analysis in the groups to which they were originally assigned.

#### Safety and acceptability

There were no safety issues such as falls during the assessments. Participants were allowed to rest at any time during instructions and assessments. The home-based intervention could be practised in a sitting position and was reported by the participants to be safe and convenient. Phone calls showed that one participant had initial difficulties with the kinaesthetic motor imagery, but these problems were reduced through practice. Two participants reported concentration problems which became better with practice. One participant in group A reported her ability to perform motor imagery decreased with increasing music complexity.

All participants in group A reported that they liked the music melodies, particularly because different music styles were used and that the beats supported their motor imagery of walking. Five out of 10 participants in group B regarded the metronome cues being helpful to keep the tempo of their imagined steps, 2/10 concerned them neutral and 3/10 found them boring. All participants in group B regarded the variety of tone pitches being acceptable. All but one of the participants (in group B) reported that the verbal cueing of the researcher facilitated their attention with the motor imagery and helped them to stay with the beat. No other participants reported problems, and 8 out of 10 participants in group A and 4 out of 10 in group B reported the intervention as being pleasurable. To summarise, the intervention was found to be acceptable despite some initial barriers which would be worthy of further investigation in the main study.

#### Adverse events and compliance

There were no reported adverse events related to this study. The intervention was home-based; therefore, the researchers had to rely on reported adherence. Participants reported to have practised median five (range four, six) times per week. Referring to a proposed practice frequency of six times per week, compliance was considered good.

#### Fatigue

As can be seen from Table 2, there was no worsening in fatigue after the interventions. Fatigue, as assessed by the MFIS total score, improved from baseline to follow-up in group A by median −9.5 (range −31, 5) points, in group B by −13 (range −28, 7) points and in group C by −3 (range −17, 4) points. Based on previous studies, the cutoff value for MS-related fatigue was set at ≥38 points on the MFIS total score [55]. Accordingly, 2 out of 10 participants in all groups A, B and C had fatigue, which was a reduction when compared to baseline with 4/10 participants with fatigue in group A, 2/10 in group B and 4/10 in group C.

**Table 2:** Follow-up data for each study group

Parameter	Group A	Group B	Group C
	Music cued motor imagery	Metronome cued motor imagery	Control group
MFIS total score (points)			
At follow-up^a^	28 (0, 53)	20 (3, 50)	29 (0, 46)
Participants with fatigue (MFIS total score ≥38)	2/10	2/10	2/10
Change to baseline^a^	−9.5 (−31, 5)	−13 (−28, 7)	−3 (−17, 4)
T25FW (seconds)			
At follow-up^b^	5.2 (3.6, 6.7)	4.5 (3.7, 5.2)	5.5 (4.5, 6.6)
Change to baseline^b^	−0.9 (−1.3, −0.5)	−0.9 (−1.3, −0.5)	0.4 (−0.3, 1.1)
Effect size^c^	1.27 (0.26, 2.17)	1.27 (0.26, 2.17)	
Participants with clinically significant improvement (≥20 %)^d^	3/10	3/10	0/10
	30 % (7, 65 %)	30 % (7, 65 %)	0 % (0)
6MWT (metres)			
At follow-up^b^	521.1 (428.8, 613.5)	521.1 (454.1, 588.0)	475.3 (384.9, 565.8)
Change to baseline^b^	68.1 (51.4, 84.7)	92.9 (55.2, 130.5)	−9.4 (−35.6, 16.9)
Effect size^c^	1.91 (0.79, 2.88)	1.72 (0.64, 2.67)	
Participants with clinically significant improvement (≥20 %)^d^	2/10	6/10	0/10
	20 % (2, 56 %)	60 % (26, 88 %)	0 % (0)

With walking speed (T25FW), improvement is indicated by a minus and worsening by a plus; with walking distance (6MWT), improvement is indicated by a plus and worsening by a minus

*T25FW* Timed 25-Foot Walk, *6MWT* 6-Minute Walk Test

^a^Median (range)

^b^Mean (95 % confidence interval)

^c^Cohen’s d, with 95 % confidence interval, based on corrected estimates of pooled standard deviation for 80 % of trials [52]

^d^Percentage (95 % confidence interval)

### Secondary outcomes

#### Walking speed

For the walking outcomes (T25FW; 6MWT), means and 95 % CI for participants in groups A, B and C at follow-up are shown in Table 2. The mean improvement in walking speed from baseline to follow-up expressed at a 95 % CI was −0.9 s (−1.3, −0.5) in groups A and B, and the mean worsening was 0.4 s (−0.3, 1.1) in group C. Based on the literature and clinical judgement, a benchmark for a clinically significant change in walking speed was set at ≥20 %. Three participants in both groups A and B but no participant in the control group C showed a clinically meaningful improvement ≥20 % from baseline to follow-up. The current potential of both interventions to induce such a clinically significant improvement in the main study is 30 % (95 % CI 7, 65 %). The two participants using walking sticks during the assessments did not show a clinically significant improvement in walking speed and distance.

#### Walking distance

The mean improvement in walking distance from baseline to follow-up expressed at a 95 % confidence interval in group A was 68.1 m (51.4, 84.7) and in group B 92.9 m (55.2, 130.5), and the mean worsening in group C was −9.4 m (−35.6, 16.9). Based on the literature and clinical judgement, a benchmark for a clinically significant change in walking distance was set at ≥20 %. Two participants in group A and six participants in group B but no participant in group C had a clinically meaningful improvement ≥20 % from baseline to follow-up. The current potential of music and metronome cued motor imagery to induce such a clinically significant improvement in the main study is 20 % (95 % CI 2, 56 %) and 60 % (95 % CI 26, 88 %), respectively.

### Implications for main study

From the feasibility and secondary outcomes, results follow that a full-scale RCT is feasible. Requirements devised for the main study are described in the next paragraphs.

#### Sample size

The estimated a priori sample size according to the study protocol was 112–150 participants, as approved by the ethics committees of the University of Brighton and Medical University of Innsbruck. On basis of the results of the walking outcomes (walking speed and walking distance), which will be the primary outcomes of the main study, the sample size for the main study was calculated using the effect sizes with 95 % CI shown in Table 2. The effect size of the walking speed (T25FW) improvement in both groups was *d* = 1.27 (95 % CI 0.26, 2.17). The effect size of the walking distance (6MWT) improvement in group A was *d* = 1.91 (95 % CI 0.79, 2.88), and in group B, it was *d* = 1.72 (95 % CI 0.64, 2.67), both based on alpha = 0.05 and 80 % power. Corrected estimates of pooled standard deviation for 80 % of trials were used for the effect size calculations, as recommended by Vickers (2003) [52]. Based on the above reported effect size of *d* = 1.27, a sample size of 11 participants per group resulted, whereas effect sizes of *d* = 1.91 and *d* = 1.72 required a sample size of 6 and 7 participants per group, respectively. In view of the large confidence intervals and based on comparable studies, we used a moderate effect size of *d* = 0.64 of the lower bound of the 6MWT improvements. The resulting sample size was 40 participants per group. Comparable motor imagery studies in people with stroke [8] and rhythmic auditory stimulation studies in people with MS [22] also found moderate effect sizes. To our knowledge, no other motor imagery or rhythmic auditory stimulation studies in people with MS were conducted that presented effect sizes.

The retention rate in our study was 100 % (95 % CI 88.6, 100 %); therefore, an attrition rate of up to 11 % might occur. Retention rates in three motor imagery studies, in a small study in people with MS were 100 % [6], and in larger studies in people with stroke were 92.6 % [8] and 85.1 % [56], respectively. Thus, our own data and comparable studies justify a conservative estimation of a 15 % attrition rate. Forty multiplied with 3 results in the number of 120. By addition of 15 % (18 participants), a sample size of 138 participants results. In order to increase power, up to 150 participants will be aimed at, according to the ethics approval.

#### Randomisation

As can be seen from Table 1, participant allocation was balanced with regard to age, disability and baseline walking but unbalanced in terms of gender. Therefore, the randomisation will be stratified on the three relevant predictive factors for a change in walking in people with MS, namely age, gender and disability (EDSS). The stratification will help ensure that the intervention and control groups are similar with respect to patient characteristics. Stratification requires restricted allocation, typically blocking which ensures a balance in sample size across groups. Therefore, stratified block randomisation in permuted fixed blocks of three with a computer random number generator will be performed (Sealed Envelope, London, UK).

#### Allocation concealment

To avoid allocation bias, allocation concealment will be performed in the main trial. Each participant will have a unique identification number (ID). A computer generated randomisation list based on the predefined strata will be produced by an independent researcher at Medical University of Innsbruck who will also create sealed opaque envelopes including group allocation numbers A, B and C. The envelopes will be stored in a sequentially numbered order based on the generated list and allocated to each participant in the order in which they are recruited. Participants will be asked to unseal the envelopes themselves and not to discuss their group allocation until study completion.

#### Blinding

In the main study, it will be worthwhile to consider blinding, for two reasons: Participants who are not aware of their group allocation might less likely show changes in behaviour, motivation and adherence. If researchers who are collecting the outcome data are not aware of the participant group allocation, they will not be influenced by their knowledge in any way. Blinding of the participants would be feasible by introduction of a placebo intervention, such as music listening. However, that would change the study design as it was not tested in the pilot study, and therefore the study data could not be used in the main study. Blinding of the researcher will be possible by involvement of a second researcher who is responsible for the outcome assessments.

## Discussion

To our knowledge, this was the first trial to investigate the impact of rhythmic cued motor imagery on walking in people with MS. Our pilot study evaluated the feasibility of the methods used for a larger RCT that will investigate the effects of rhythmic cued motor imagery on walking in people with MS. For this purpose, the success of the feasibility criteria recruitment rate, duration of recruitment, retention rate, safety, adverse events, compliance, acceptability of the interventions and fatigue was assessed. It was evaluated whether music or metronome cued motor imagery negatively impacted on fatigue which would have led to dropping one of the intervention arms. In addition, the group differences between baseline and follow-up on the secondary outcomes in walking speed and walking distance were measured. The differences in walking speed and walking distance was used to recalculate the sample size for the main study.

We achieved our recruitment target by recruiting 12 participants per month, an eligibility rate of 40.1 % out of 2500 MS Centre patients, a consent rate of 15.9 % and additional 54.5 % of eligible patients who expressed their interest to participate. Following from this, approximately 12 months are estimated for the main study recruitment, a time period which is well below our maximum available time period of 20 months. The retention rate in our study was 100 %, compared to motor imagery studies with retention rates of 100 % in people with MS [6], as well as 92.6 % [8] and 85.1 % [56] in people with stroke. As we cannot expect to have a 100 % retention rate in a larger trial, we based the sample size calculation on an 85 % retention rate.

There were no safety issues during the assessments and no adverse events associated with the interventions. Participants considered the rhythmic cued motor imagery interventions to be safe and convenient because they could practise while seated in their homes. Eight out of 10 participants in the music cued motor imagery group, and 4 out of 10 in the metronome cued motor imagery group regarded the intervention to be pleasurable. This result is not surprising as metronome cues are monotonous, whereas music melodies and rhythms carry emotion and are known to have an influence on mood, the desire to move and work output. In the main study, additional quality of life measures will be used to assess the impact of the music and metronome cued motor imagery on psychological functioning. The majority of participants reported the music and metronome cueing being helpful to keep the tempo of their imagined steps and the verbal cueing being supportive to maintain their attention and stay with the rhythm; thus, the same cueing types will be used in the interventions of the main study. The overall compliance was good with a practice frequency per week of median 5 (range 4, 6). We expect a similar compliance in the main study, in particular as the interventions were acceptable, if not pleasurable to participants.

The intervention of our study was home-based which might have had an impact on adherence, in two respects: first, participants were responsible to comply with the practice frequency of six times a week for 4 weeks as it would not be practical to supervise on a 6× week basis. We had to rely on the participants’ reports of their adherence which was noted by the researcher during weekly phone calls and follow-up assessments. Secondly, the setting seemed to be beneficial insofar as the participants were not required to travel for the intervention, as it is known that many people with MS have difficulties with driving a car and walking.

We observed a mild improvement in fatigue in all groups at follow-up when compared to baseline. This means that none of the interventions worsened fatigue so that both the music and metronome cued motor imagery arm will be used in the main study. Interestingly, fatigue was also slightly improved in the control group, probably due to motivational aspects related to being in a study.

Motor imagery with instrumental music as well as motor imagery with metronome cues improved walking speed and walking distance in the intervention groups compared to controls. From a patient viewpoint, an improvement in walking speed is only useful if it reaches clinical significance, because only in such a case the intervention benefits the person in their activities of daily living. Recent studies showed that a ≥20 % change in walking speed exceeds the day-to-day differences in participants’ walking performance and the variability of the measurements, and therefore, such a change can be considered a clinically meaningful change [42, 43]. Indeed, three participants in our study in both groups A and B showed a clinically significant improvement in walking speed which is consistent with a current 30 %(95 % CI 7, 65 %) potential for such an improvement in the main study.

Rhythmic cued motor imagery also improved walking distance as measured by the 6MWT in participants of both intervention groups when compared to the control group. Learmonth et al. found that a ≥20 % improvement might be regarded clinically meaningful [43]. Such an improvement might enable persons to maintain their independence and to increase their range of activities, which might influence their quality of life and their ability to work. In our study, two participants in group A and six participants in group B reached a clinically significant improvement in walking distance, consistent with a current potential of intervention A to induce such an improvement in the main study of 20 % (95 % CI 2, 56 %) and in group B of 60 % (95 % CI 26, 88 %). In contrast to our study, in their large multicentre study, Baert et al. reported a change of 10 % (21.6 metres) in the 6MWT measures to be sufficiently large for a clinically meaningful improvement [47]; however, their study population had more severe disability.

Based on our clinical experience, our suggestions were that the participants might benefit more from the rhythmic cued motor imagery if they would be able to truly understand what to practice. Hence, our participants were thoroughly informed on the motor imagery and rhythmic cueing. Our approach was in line with other studies who explicitly introduced participants to the motor imagery theory and to the perspective to be adopted during the intervention [57, 58]. A motor imagery familiarisation was carried out similar to that proposed by Wondrusch and Schuster-Amft in 2013 [34].

Our strategy to facilitate motor imagery was to add external rhythmic auditory cues, which has been effective in other studies [59, 23]. Besides music and metronome cues, rhythmic verbal cueing, in accordance with the cueing tempo, was used to accentuate the timing structure of the rhythmic cued motor imagery. A similar approach had also been proposed by the recent literature on the use of patterned sensory enhancement for facilitation of complex movements in people with neurologic diseases including MS [15, 14]. The additional cueing might have supported the participants to practise the motor imagery, and the verbal cueing might have helped to maintain the temporal patterns of walking during the motor imagery [60].

As far as we know, our participants were able to perform motor imagery. Our results seem to be in contrast to previous studies demonstrating a lower capacity for motor imagery in people with MS [61, 12]. However, these authors linked impaired motor imagery in this population particularly to cognitive dysfunction [61, 12] and depression [62]. Therefore, persons with cognitive impairment and depression were excluded from our study. Several studies used patient-rated questionnaires, such as the Kinaesthetic and Visual Imagery Questionnaire to assess the motor imagery ability in their participants [12, 62]. Our study could have used this patient-rated questionnaire, but our participants were called weekly to ask for any problems with kinaesthetic motor imagery, and they were supported accordingly. In addition, all motor imagery ability studies in people with MS were experimental studies with no long-term training effects, in contrast to our 4 weeks duration study with 24 training sessions which might have enhanced the mental representation [63].

This study was conducted as a single-centre study, which might be an advantage because the study was implemented in the same way with all participants. Only outcomes that are reliable and valid in the MS population were used. Application of these measures facilitates comparisons with other studies and the possibility of a meta-analysis at a later point in time.

There are several possible limitations to this pilot study. Participants in all groups including the control group were called weekly, asking about their health condition. This means we spent time on the participants of the control arm and accompanied them over the 4 weeks study period, which probably contributed to their improvement in fatigue. Nevertheless, participants of the intervention groups received additional motor imagery familiarisation and the researcher’s instructions and verbal cueing on the CDs. The extra attention paid to participants in groups A and B might have increased their motivation to walk faster and a larger distance. However, after study completion several participants decided to continue their practice since their walking had improved to such a great extent. The study was underpowered because of the small sample size, but this was a pilot study to enable us to calculate the required sample size for the main trial. The results are to be considered preliminary, and they cannot be generalised yet to a larger population of people with MS. A subsequent well-powered main study is being conducted.

There was an imbalance of groups with respect to gender. To ensure a balanced group allocation in the main study, the randomisation will be stratified on the predictive factors gender, age and disability. To guarantee a balanced group size, block randomisation in permuted blocks will be used. Another possible limitation of the study is the use of a single physiotherapist to give instruction to participants and undertake all measurements. To overcome this limitation, a script was used and all instructions and support were carried out consistently. Finally, the lack of blinding is another limitation and is a challenge in physiotherapy trials. However, blinding of the participants would not have been possible as they would have realised their group allocation. It is unlikely that blinding of the researcher would have made any difference to the findings as both walking tests were performed according to internationally recognised guidelines and instructions. However, it is advisable to implement blinding of the researcher in the main study which can be done by employment of a second researcher responsible for the assessments. The randomisation procedure should have made allocation concealment redundant as the researcher could not influence participant allocation to groups. To prevent possible allocation bias in the main study, allocation concealment will be performed.

## Conclusions

The results from our pilot study showed that a larger main study is feasible to investigate the effects of rhythmic cued motor imagery on walking, fatigue and quality of life in people with MS. Success of the feasibility criteria demonstrated that no changes to the interventions and assessments will be required. Based on the walking improvements, a sample size of 46 participants in three groups, that is a total sample size of 138 participants by inclusion of 15 % attrition, was calculated for the main study. To avoid bias, stratified blocked randomisation, allocation concealment and blinding will be used. The preliminary improvements in walking speed, walking distance and fatigue need to be confirmed in a larger trial.

## Abbreviations

6MWT: 6-Minute Walk Test
AKM: Austrian State Authorized Society of Authors, Composers and Publishers
CD: compact disc
ECTRIMS: European Committee for Treatment and Research in MS
EDSS: Expanded Disability Status Scale
MS: multiple sclerosis
T25FW: Timed 25-Foot Walk
